# Alternatives to conduits

**DOI:** 10.4103/0974-2069.41055

**Published:** 2008

**Authors:** Krishna S. Iyer

**Affiliations:** Department of Pediatric and Congenital Heart Surgery, Escorts Heart Institute and Research Centre, New Delhi, India

Surgery for congenital heart disease has progressed by leaps and bounds in the last few decades, but the right ventricular outflow tract continues to pose a challenge to the congenital heart surgeon. A sizeable proportion of congenital heart defects have a component of right ventricular outflow tract abnormality. This may be in the form of a simple stenosis or a more complicated atresia, discordant ventriculo-arterial connection, absent pulmonary valve or rarely a common systemic and pulmonary outflow as in truncus arteriosus. Pulmonary stenosis (PS) is relatively easily treated by opening the stenosed pulmonary valve, resecting obstructive muscle and if necessary further enlarging the narrowed portion with a patch of autologous pericardium. Since a portion of the natural right ventricle (RV) to pulmonary artery (PA) connection is preserved, the outflow tract can be expected to enlarge in keeping with the growth of the patient and recurrent stenosis is rather uncommon.

Absence of continuity between the RV and PA either because of atresia or a discordant arterial connection calls for a more complicated intervention. Valved conduits were first used by Ross[1] and soon after by Rastelli[2] in the early 1960's, and since then have remained the mainstay of the treatment of RV - PA discontinuity. Valved conduits do a wonderful job of mimicking the natural right ventricular outflow, however they have one major drawback - they do not grow! This means that once a patient receives a conduit, re-operation for conduit replacement is inevitable. Growth may not be a relevant issue in the older patient who has attained full physical development, however, conduit stenosis warranting replacement invariably develops as a result of intimal peel formation, anastamotic stricture or calcific degeneration of the conduit valve.[3] Nonetheless conduits have been known to function satisfactorily for upwards of ten years in the older patient. When used in neonates and young children however, conduit longevity is markedly shortened because of a combination of progressive body-weight / conduit size mismatch and a poorly understood accelerated degeneration of the conduit valve.[4] In this age group conduit replacement may be required within a few months of implantation.

A wide range of conduits are currently in use - cryopreserved or fresh antibiotic sterilized aortic and pulmonary homografts, dacron tubes with porcine aortic valves, bovine pericardial tubes with porcine aortic valves, porcine aortic roots and more recently bovine internal jugular vein. Most non-homograft conduits are commercially available in most parts of the world in a wide range of sizes. Homografts are largely harvested and prepared by individual institutions in valve banks that are specially set-up for this purpose. Availability of homografts is dependant on the supply of cadaver hearts and is generally limited, in the smaller sizes. However, when available they are the preferred conduit of choice because of their superior handling characteristics and longevity. Commercially available conduits overcome the problem of availability but are generally prohibitively expensive, precluding their widespread use in countries with meager financial resources. Surgeons often resort to handmade conduits made in the operating room from autologous or bovine pericardium, dacron or polytetrafluoroethylene (PTFE).[5,6] The search for the ideal conduit that not only resists degeneration but also grows commensurate with the needs of the patient continues, but such a conduit remains elusive. Tissue engineered conduits derived from autologous cell cultures hold some promise, but, as of now, have not entered the clinical arena.[7]

Given the problems with the use of conduits, it is not surprising that surgeons have constantly endeavored to evolve surgical techniques that would obviate their use. The common surgical principle in all these so called ‘non-conduit options’ is: 1) direct autologous tissue approximation of the opened out main PA or PA bifurcation to the superior margin of the right ventriculotomy, thus creating the posterior wall of the RV outflow, and 2) roofing this with a generous patch of autologous pericardium to create the anterior wall of the outflow. The assumption is that, as in correction of tetralogy of Fallot with a transannular outflow patch, there is a direct continuity of right ventricular muscle with native PA along, at least some part of the circumference of the reconstructed outflow and this would allow age-related growth. Additionally, some attempt is made at containing severe pulmonary regurgitation by incorporating a monocusp made of autologous pericardium, homograft cusp, bovine pericardium or PTFE membrane. However, unlike conduits the competence of the monocusp valve is dependant on the surgeon's skill.

Conduitless repair is most easily performed in patients with ventricular septal defect (VSD) and short segment pulmonary atresia. Here the gap between the RV outflow and the PA is short, and approximation of the mobilized main pulmonary artery stump to the right ventriculotomy is relatively easily achieved without too much tension on the PAs.[8] Approximation is not easy when the gap is more as in transposition and may result in excessive stretch of the branch PAs or compression of the coronary arteries at their origin. Anterior translocation of the pulmonary artery bifurcation (the Lecompte maneuver) is often performed to overcome this problem.

The first of these types of repair for transposition with VSD and PS was described by Lecompte in 1982 and he named the operation ‘Reparation L'etage Ventriculaire’ or REV procedure in short.[9] The left ventricle is routed to the aorta with an intracardiac patch through a right ventriculotomy, as in the conventional Rastelli operation with the difference that the infundibular septum is generously resected to provide a straighter path from the left ventricle to aorta. This is aimed at reducing the possibility of late left ventricular outflow obstruction. The main PA is then transected at the level of the stenotic pulmonary valve and the proximal stump is closed. The ascending aorta is then transected to allow anterior translocation of the PA bifurcation and then reanastamosed behind the PA. The PA branches need to be mobilized extensively into the lung hilum on either side to allow a comfortable lie in front of the ascending aorta. A wedge-shaped segment of the ascending aorta is often excised to reduce the stretch on the PA bifurcation. The posterior wall of the opened out main PA stump is then sutured to the upper margin of the right ventriculotomy. A monocusp made of glutaraldehyde treated autologous pericardium is sutured to the right ventriculotomy and the outflow reconstruction is completed with a hood of native pericardium that covers the ventriculotomy and the main PA orifice.

Although the REV procedure is a theoretically attractive procedure that obviates the use of a conduit it is not feasible in all patients with discordant ventriculo-arterial connection. Patients who have had prior systemic to pulmonary shunts tend to have fibrosis and thickening of the PAs that makes the Lecompte maneuver difficult or unsafe. In patients with a large ascending aorta as might be seen in transposition with pulmonary atresia, there can be excessive stretching of the PA bifurcation leading to outflow obstruction. In patients with a limited retrosternal space or in those with pectus excavatum, the anteriorly placed pericardial hood is at risk of compression.

In 1984, Nikaidoh further extended this principle in his technique for non-conduit repair of transposition with VSD and PS.[10] In his technique the left ventricular outflow is further straightened by excising the aortic root from the right ventricular outflow (in much the same way as the pulmonary autograft is harvested in the Ross procedure) and suturing it to the left ventricular outflow after excision of the pulmonary root. This posterior translocation of the aortic root often necessitates explantation and relocation of one or both coronary arteries as well. The VSD is closed with a patch to complete the left ventricular outflow following which the RV outflow is then reconstructed in much the same way as in the REV procedure, after performing the Lecompte maneuver. The Nikaidoh procedure is more elaborate and challenging than the REV procedure but is more anatomic and produces less stretch on the pulmonary arteries because of the posterior displacement of the ascending aorta. As with the REV procedure, the Nikaidoh procedure is also not feasible in all patients of transposition with VSD and PS. Important coronary artery crossing the RV outflow, pulmonary atresia or remote location of the VSD are situations where the Nikaidoh procedure would be inadvisable. However, even when anatomic variations preclude the use of the REV procedure or the Nikaidoh procedure in their original form surgeons can use modifications and combinations of these two procedures to achieve a conduitless repair. With increasing experience these techniques have now been further adapted to the treatment of other conotruncal lesions like double outlet right ventricle with malposed great vessels and corrected transposition with VSD and PS.[11]

Early attempts at conduitless RV-PA connection were confined to conditions with decreased pulmonary blood flow, because it was feared that in patients with increased pulmonary blood flow, postoperative pulmonary incompetence in the face of pulmonary hypertension would not be well tolerated and lead to adverse outcomes. The first successful attempt at non-conduit repair for truncus arteriosus was reported by Reid in 1986.[12] Subsequently a series of cases were reported by Barbero-Marcial.[13] In his original description of the technique for Type I and Type II lesions the truncus was septated with a patch and the VSD closed to create a valved systemic outflow. The pulmonary component of the truncus was then anastamosed to the right ventriculotomy using a flap of the left atrial appendage to bridge the gap and create the posterior wall of the RV outflow. The anterior wall was then completed using autologous pericardium after a monocusp valve had been sutured to the right ventriculotomy. Subsequent modifications by others have attempted to avoid the use of the left atrial appendage in the posterior wall.

Although conduit sparing procedures have now been in use for over two decades, long-term data in large groups of patients is lacking, unlike follow-up data for the Rastelli procedure.[3,14] Meaningful comparisons between the two procedures is therefore difficult. Some reasonable inferences may be drawn from currently available literature. Early survival in patients with decreased pulmonary blood flow is comparable between the two groups.[11] The REV procedure and the Nikaidoh procedure offer improved long-term freedom from left ventricular outflow obstruction as compared to the conventional Rastelli procedure in transposition of great arteries with VSD and PS.[15,16] Re-intervention for RV outflow obstruction is probably reduced with non-conduit surgery but is certainly not insignificant.[16,17] Of concern, is the fact that most of these patients develop significant pulmonary regurgitation over time, as the monocusp becomes non-functional. The detrimental effects of chronic pulmonary regurgitation on RV function are now becoming more evident as long-term follow-up data in patients operated for tetralogy of Fallot is becoming available.[18] Chronic pulmonary regurgitation is likely to have more deleterious effects in these patients because in comparison with patients of tetralogy of Fallot they have a larger ventricular incision which tends to be more in the body of the RV rather than in the infundibulum. It is likely that with longer follow-up these patients will require pulmonary valve implantation for preservation of RV function. Clearly, the question that needs to be answered is whether with conduitless surgery, we are paying for freedom from reintervention with a greater risk of long-term RV dysfunction.

In truncus arteriosus, it appears that beyond the neonatal period non-conduit repairs seem to be associated with a greater early mortality,[19] possibly because of the higher incidence of postoperative pulmonary hypertension. Favorable results have been demonstrated in neonates, however, where even totally valveless tubes have yielded outcomes comparable to conduit repairs.[20,21] In neonates, tissue friability and limitations of space make conventional conduit repair difficult, and therefore non-conduit options hold greater appeal. As with low pulmonary blood flow situations, the concerns about long-term RV dysfunction remain.
